# Effect of the fluorination technique on the surface-fluorination patterning of double-walled carbon nanotubes

**DOI:** 10.3762/bjnano.8.169

**Published:** 2017-08-15

**Authors:** Lyubov G Bulusheva, Yuliya V Fedoseeva, Emmanuel Flahaut, Jérémy Rio, Christopher P Ewels, Victor O Koroteev, Gregory Van Lier, Denis V Vyalikh, Alexander V Okotrub

**Affiliations:** 1 Nikolaev Institute of Inorganic Chemistry, SB RAS, 630090 Novosibirsk, Russia; 2 Novosibirsk State University, 630090 Novosibirsk, Russia; 3 Centre Interuniversitaire de Recherche et d’Ingenierie des Materiaux, Universite Paul-Sabatier, F-31062 Toulouse 9, France; 4 CNRS, Institut Carnot Cirimat, F-31062 Toulouse, France; 5 Institut des Materiaux Jean Rouxel, CNRS-Université de Nantes, F-44322 Nantes, France; 6 Vrije Universiteit Brussel – Free University of Brussels (VUB), Pleinlaan 2, B-1050 Brussels, Belgium; 7 Donostia International Physics Center, Departamento de Fisica de Materiales and CFM-MPC UPV/EHU, 20080 San Sebastian, Spain; 8 IKERBASQUE, Basque Foundation for Science, 48011 Bilbao, Spain

**Keywords:** double-walled carbon nanotubes, fluorination, NEXAFS, quantum-chemical modeling

## Abstract

Double-walled carbon nanotubes (DWCNTs) are fluorinated using (1) fluorine F_2_ at 200 °C, (2) gaseous BrF_3_ at room temperature, and (3) CF_4_ radio-frequency plasma functionalization. These have been comparatively studied using transmission electron microscopy and infrared, Raman, X-ray photoelectron, and near-edge X-ray absorption fine structure (NEXAFS) spectroscopy. A formation of covalent C–F bonds and a considerable reduction in the intensity of radial breathing modes from the outer shells of DWCNTs are observed for all samples. Differences in the electronic state of fluorine and the C–F vibrations for three kinds of the fluorinated DWCNTs are attributed to distinct local surroundings of the attached fluorine atoms. Possible fluorine patterns realized through a certain fluorination technique are revealed from comparison of experimental NEXAFS F K-edge spectra with quantum-chemical calculations of various models. It is proposed that fluorination with F_2_ and BrF_3_ produces small fully fluorinated areas and short fluorinated chains, respectively, while the treatment with CF_4_ plasma results in various attached species, including single or paired fluorine atoms and –CF_3_ groups. The results demonstrate a possibility of different patterning of carbon surfaces through choosing the fluorination method.

## Introduction

Even after surface chemical functionalization, due to their inner shell double-walled carbon nanotubes (DWCNTs) display many advantages characteristic of single-walled carbon nanotubes (SWCNTs), particularly small diameter, high strength and flexibility [1]. Carbon nanotube (CNT) surfaces are rather inert to chemical functionalization. The highest possible concentration of attached surface species is achieved through fluorination because fluorine is the most electronegative element and highly reactive, while its small atomic radius compared to other functional groups allows potentially high density surface packing. The maximal C_2_F ratio for SWCNTs is obtained using elemental fluorine at 200–300 °C [2]. The application of a similar fluorination procedure to DWCNTs yields a product with overall CF_0.3_ composition, leaving the inner shells intact [3]. The higher fluorination loading, obtained through an increase of the synthesis temperature, creates defects in the DWCNT surface and introduces fluorine onto the inner shell too [4].

Although fluorinated CNTs are generally expected to be insulating, one-dimensional structures with a conducting shell surrounded by an insulating layer from the fluorinated carbon could find potential application in nanoelectronics and gas sensing [5]. The ability to change the functional composition of the outer shell would significantly extend the areas of possible DWCNT applications. For example, quantum-chemical calculations predict that the conductivity of fluorinated CNTs changes from semiconducting to metallic depending on surface distribution of fluorine atoms [6]. Furthermore, the energy of a C–F bond decreases with reduction of fluorine content in CNTs [7], which should promote nucleophilic substitution reactions, leading to new derivatives [8–9]. Fluorinated CNTs have a potential in chromatographic separations of various halogenated compounds owing to an optimal combination of hydrophobic properties and specific polar interactions [10]. The promises of the fluorinated CNTs may be fully realized only when the fluorine atoms would be controllably attached to the nanotube surface and the search of the appropriate ways for that is one of the key points in this scientific field at present [11].

There are several ways to fluorinate CNTs, the most common being fluorination using F_2_ gas [12], CF_4_ plasma [13], and BrF_3_ vapor [14]. For all of these methods, the parameters preserving the tubular structure of the nanotubes after the fluorination have been determined. The high thermal stability of F_2_ means elevated temperatures are required in order for the fluorination process to take place. The saturation composition C_2_F for a CNT surface was achieved using pure F_2_ below 300 °C for several hours [15]. The temperature and/or duration of the synthesis can be reduced substantially in the presence of HF, which catalyzes the breaking of the F–F bond [16]. The non-metallic fluorides are much more reactive than elemental fluorine, and can interact with the graphitic surface even at room temperature [17]. To control the energy release associated with fluorination, BrF_3_, in particular, is mixed with Br_2_ [18]. Depending on the structure of the CNT samples diffusion of the diluted vapors occurs at different rates leading to a different fluorine loading [19]. In the process of radio-frequency (rf) plasma fluorination, the rf power, gas flow rate, and exposure time should be carefully chosen to avoid nanotube damage [20].

Previously, we have revealed that the thermal behavior of the fluorinated DWCNTs strongly depends on the fluorination method [21]. An observation of fluorine removal within different temperature intervals has pointed on a difference in the bonding strength between fluorine and DWCNT surface realizing through different methods. This resulted in fluorine loss together with carbon accompanied by partial surface etching of the DWCNTs fluorinated by F_2_ and BrF_3_, while no detectable wall destruction occurred for the plasma-fluorinated DWCNTs.

We show here that the C–F bond strength is sensitive to surroundings in the addition pattern, which can be controlled through the fluorination method. The preferable fluorine distributions on the DWCNT surface are proposed from quantum-chemical modelling of the fluorine near-edge X-ray absorption fine structure (NEXAFS) spectra, which showed substantial differences for the samples prepared using elemental F_2_ at elevated temperature, BrF_3_ at room temperature, and CF_4_ rf plasma. Infrared (IR) spectroscopy and X-ray photoelectron spectroscopy (XPS) are invoked to support the proposed fluorine distributions.

## Results and Discussion

**Figure 1:**
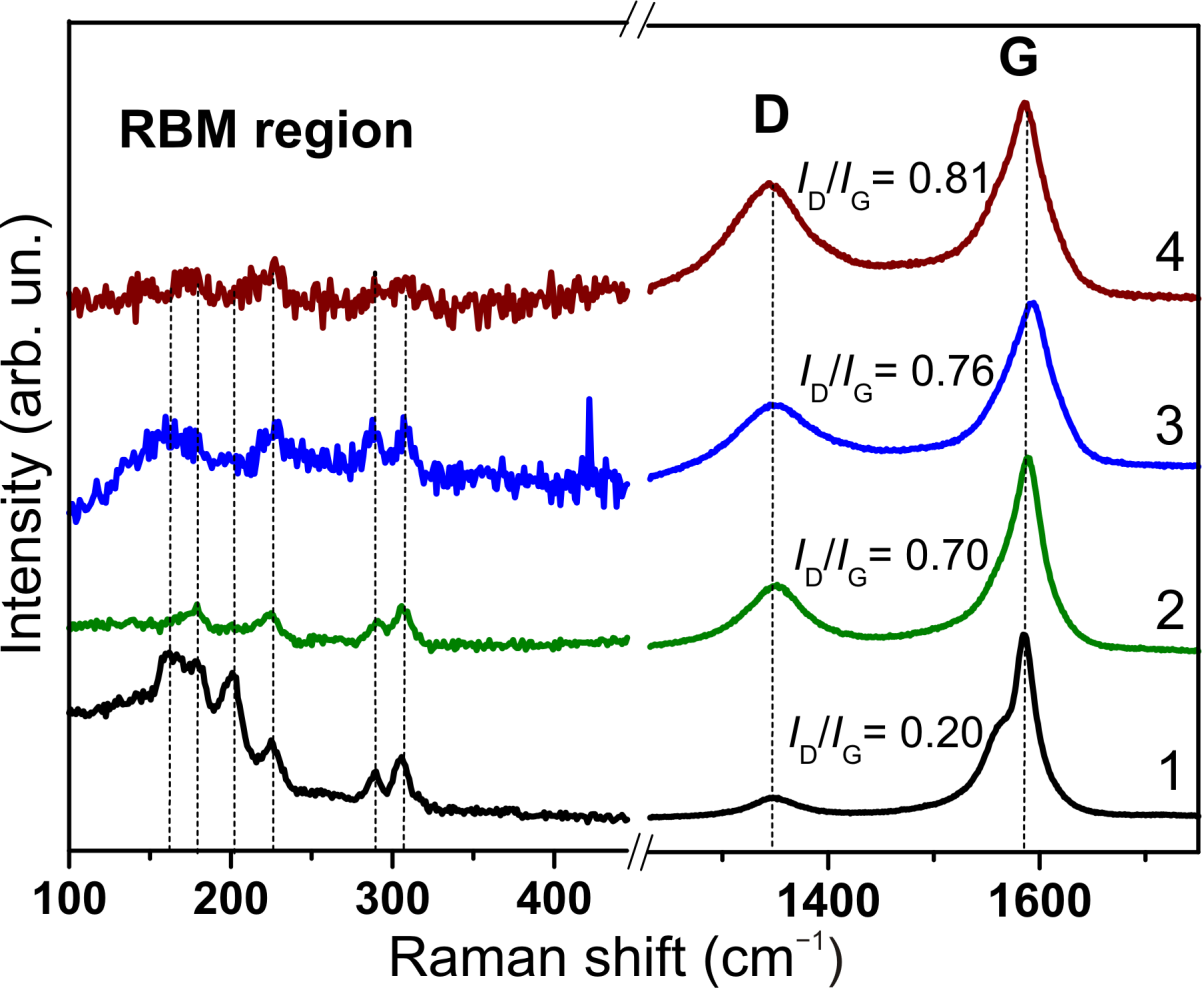
Raman spectra of pristine DWCNTs (1) and DWCNTs fluorinated with CF_4_ plasma (2), BrF_3_ (3), and F_2_ (4).

Raman spectroscopy detected a growth of the intensity of the D band corresponding to out-of-plane vibrations of carbon hexagons after fluorination of the DWCNT sample (Figure 1). This is due to development of sp^3^-hybridized carbon defect sites as the result of covalent attachment of fluorine to the DWCNT shells. The ratio of integral intensities of D band to G band (*I*_D_/*I*_G_) progressively grows in a sequence of the fluorination techniques (CF_4_ plasma < BrF_3_ gas < F_2_ gas), which can be attributed to an increase of the fluorine loading.

In the low-frequency region (100–400 cm^−1^) the Raman spectrum of the pristine sample exhibits two groups of radial breathing modes (RBM) (Figure 1, curve 1). The RBM peaks below ca. 250 cm^−1^ are usually attributed to the outer DWCNT shells, while the RBM peaks above ca. 250 cm^−1^ are assigned to the inner DWCNT shells [22]. Since the sample contains about 20% of SWCNTs [23], they also can contribute in the RBM region. The used CCVD procedure yields a variety of nanotube configurations, which can be identified from Raman spectra measured at different laser lines. After fluorination, the intensity in the RBM region decreases and becomes negligible in the spectrum of DWCNTs fluorinated by F_2_ (Figure 1, curve 4). This is possibly due to fluorine penetration between the DWCNT layers. However, in the spectra of DWCNTs fluorinated by CF_4_ plasma and BrF_3_ two lines are clearly visible in the range of 270–320 cm^−1^ , which can undoubtedly be attributed to the non-fluorinated inner shells of DWCNTs.

**Figure 2:**
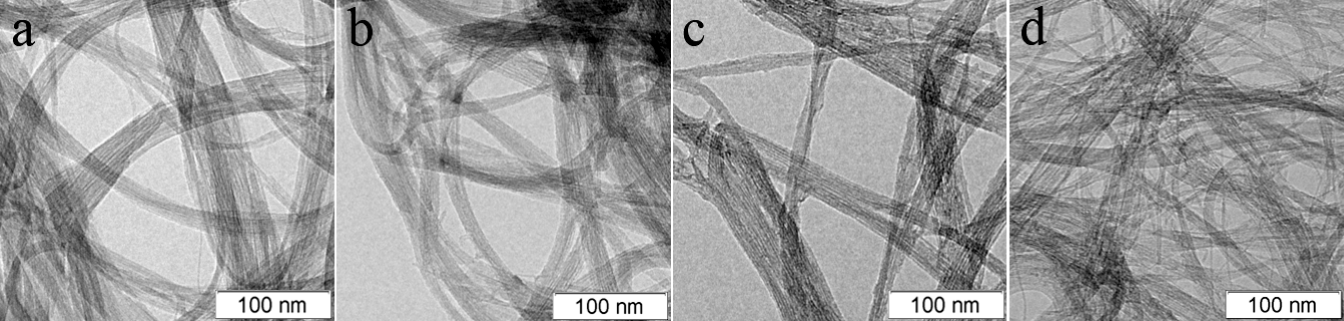
TEM images of pristine purified DWCNTs (a) and DWCNTs fluorinated with CF_4_ plasma (b), BrF_3_ (c), and F_2_ (d).

TEM analysis of the pristine and fluorinated DWCNTs revealed a different effect of the used treatments on the sample microstructure (Figure 2). The CCVD synthesis produces DWCNTs gathering into ropes with an average size of ca. 20 nm. After treatment with BrF_3_ or F_2_, this size decreases to about 10 nm and, moreover, in the latter sample many nanotubes are individual or combined into thin ropes (Figure 2d). CF_4_ plasma treatment does not result in nanotube separation (Figure 2b). Repulsion of fluorine atoms attached to the walls of neighboring nanotubes induces a splitting of ropes especially during sonication in a solution [24], which was done for the preparation of TEM specimens. The higher degree of splitting achieved for the F_2_-fluorinated DWCNTs may testify a higher fluorination yield of this technique.

**Figure 3:**
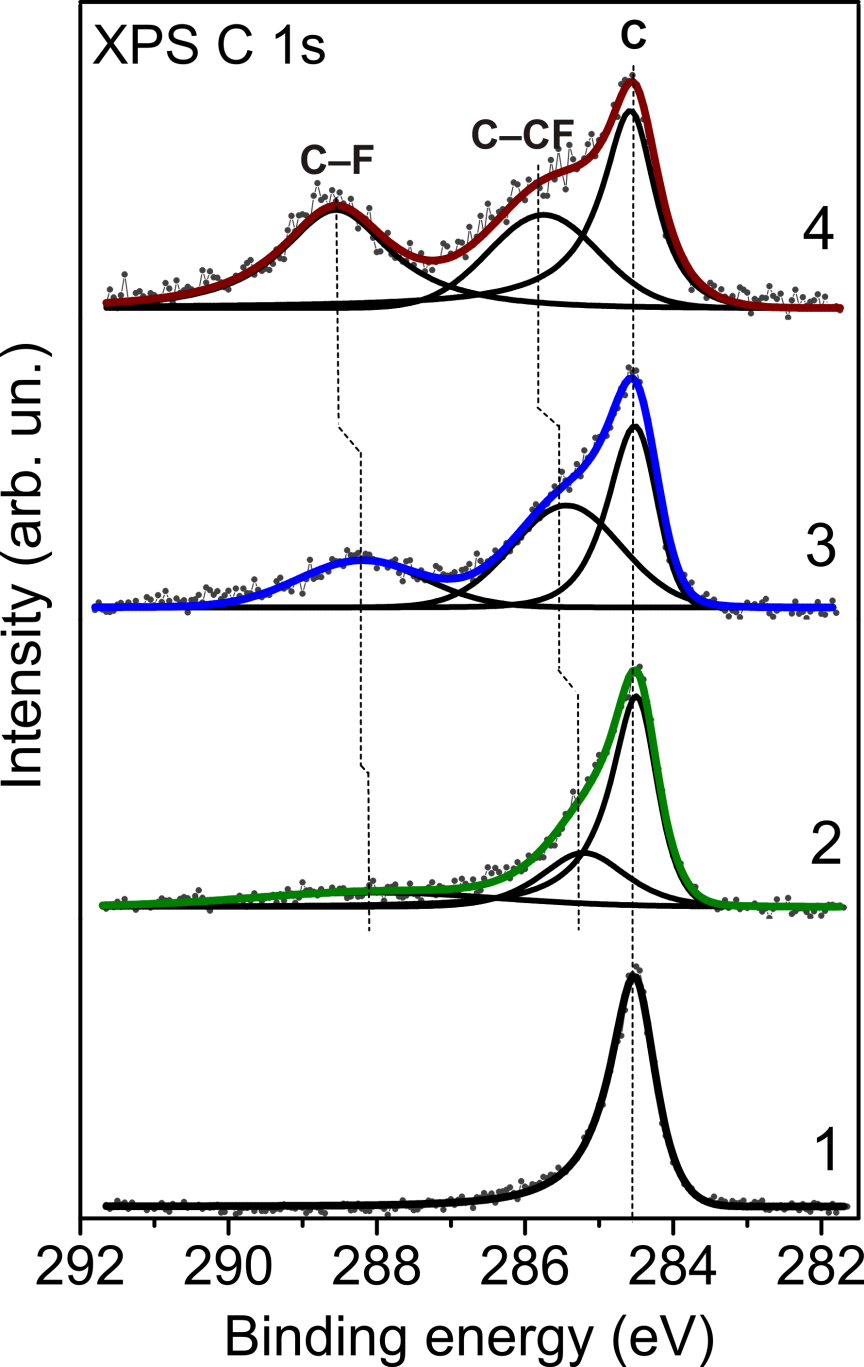
XPS C 1s spectra for pristine DWCNTs (1) and DWCNTs fluorinated with CF_4_ plasma (2), BrF_3_ (3) and F_2_.

XPS C 1s spectra detected changes in the chemical state of carbon after the DWCNT fluorination (Figure 3). Compared to the spectrum of the initial sample, the spectra of the fluorinated samples exhibit an enhanced intensity in the ranges of 285.2–285.7 eV and 288.0–288.5 eV. The latter is associated with covalent C–F bonds, while the former one corresponds to carbon atoms located next to CF groups [25–26]. The spectra were fitted using a combination of three components with a Gaussian–Lorentzian peak shape with a Doniach–Sunjic high-energy tail [27]. The integral intensities of the components were used to estimate the sample composition (Table 1). The highest surface coverage with fluorine occurs when F_2_ gas is used as a fluorinating agent. The composition of the sample, CF_0.33_, is close to that determined in [3]. But in our case the exposure time was significantly shorter (10 min versus 5 h), due to a catalytic effect of HF present in the fluorine gas [16]. The CF_4_ plasma treatment resulted in the lowest fluorination degree. The XPS C 1s spectra of the fluorinated samples show different energy positions for the CF component as well as for the C–CF component (Table 1). These components gradually move away from the sp^2^-hybridized carbon component with increased fluorine loading. This observation fully agrees with the prediction of C 1s peak separations made using the quantum-chemical calculations of fluorinated CNT models with C_2_F, C_3_F, and C_4_F compositions [28]. A ratio of the areas under the C–CF and C–F components (Table 1) gives an average number of bare carbon atoms per CF group. The number grows from 1 to 2 to 3, respectively, when F_2_, BrF_3_, and CF_4_ plasma is used as fluorinating agent. Based on these results, we suppose distinct fluorine patterns in the DWCNTs fluorinated by different techniques.

**Table 1:** Composition (CF*_x_*) of fluorinated DWCNT samples, energy positions (eV) of components of XPS C 1s spectra, and ratio of integral intensities of the C–CF and C–F components.

fluorinating agent	composition	*E*_C_	*E*_C–CF_	*E*_C–F_	*S*_C–CF_/*S*_C–F_

CF_4_	CF_0.17_	284.5	285.2	288.0	3:1
BrF_3_	CF_0.22_	284.5	285.4	288.2	2:1
F_2_	CF_0.33_	284.5	285.7	288.5	1:1

**Figure 4:**
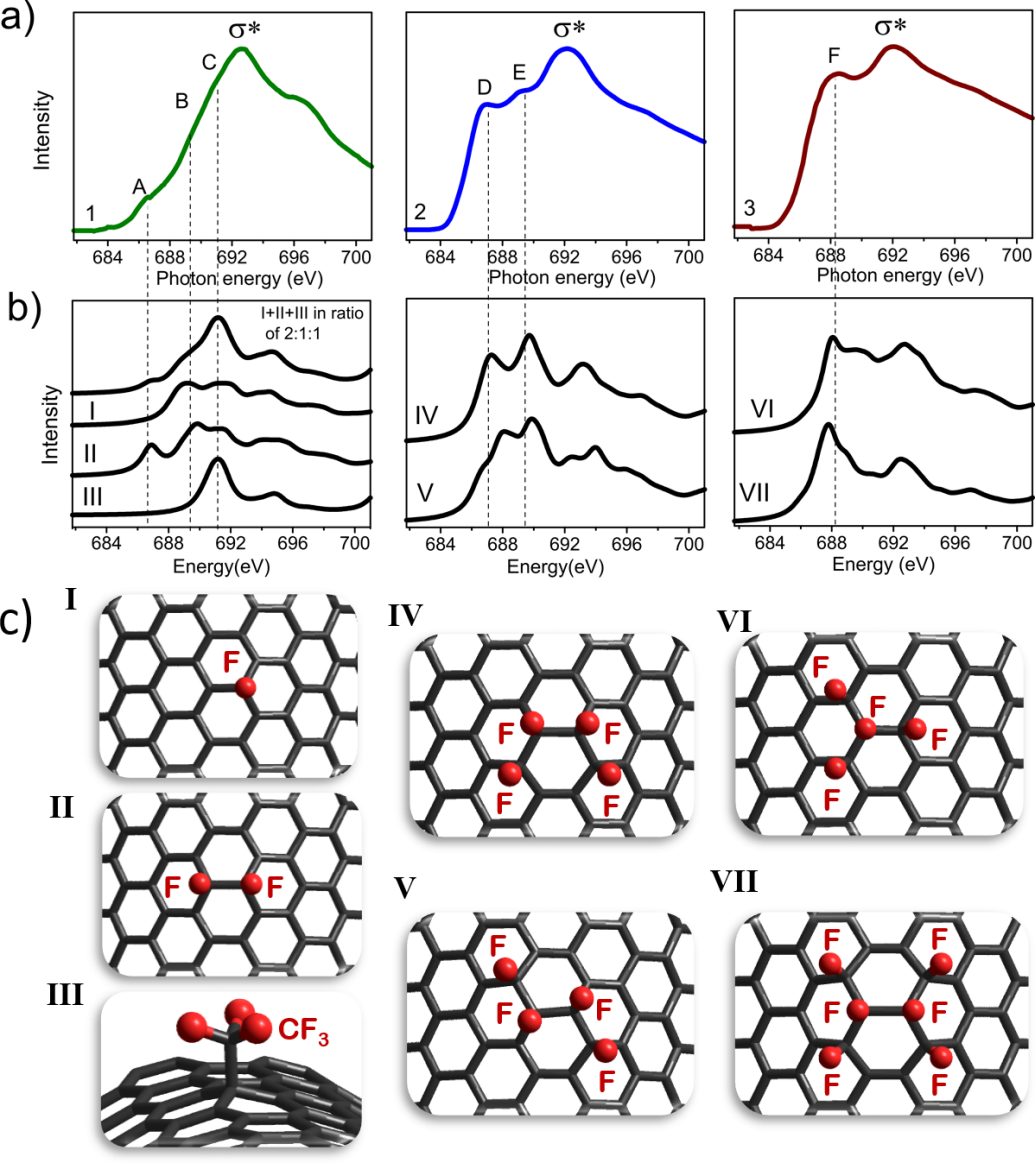
(a) Experimental F K-edge NEXAFS spectra for DWCNTs fluorinated with CF_4_ plasma (1), BrF_3_ (2) and F_2_ (3). (b) Theoretical F K-edge spectra calculated for all F atoms in models (c) distinguished by fluorination pattern. The curve above the theoretical spectra plotted for models I, II and III is their combination in a ratio of 2:1:1.

To reveal a dominating pattern of fluorine addition on the DWCNT surface during a particular fluorination procedure, we carried out simulations of the NEXAFS spectra of the fluorinated samples. NEXAFS spectroscopy is widely used for probing the surface chemical functionalities and the electronic structure of CNTs and related nanomaterials [29]. A spectrum arises as a result of core-level electrons being excited into partially filled and empty states, thus providing information about the unoccupied density of states of the X-ray absorbing elements. We consider the F K-edge spectra because they showed a considerable variation of the pre-edge features depending on the fluorination method [21]. Actually, at energies lower than those of the σ*-adsorption edge, the spectrum of plasma-fluorinated DWCNTs has a weak peak A at ca. 686.9 eV and shoulders B and C at ca. 689.6 and ca. 691.3 eV (Figure 4a, curve 1). The spectrum of DWCNTs fluorinated with BrF_3_ exhibits two peaks D and E at ca. 687.1 and ca. 689.5 eV with almost equal intensities (Figure 4a, curve 2), whereas the spectrum of DWCNTs fluorinated with F_2_ is dominated by a peak F around 688.3 eV (Figure 4a, curve 3). Features A and B observed in the F K-edge spectrum of plasma-fluorinated DWCNTs have energies close to peaks D and E, respectively, in the spectrum of BrF_3_-treated DWCNTs. However, the origin of the peaks from each pair may be different. Earlier, we have suggested that the low-energy features correspond to the interaction of fluorine with carbon atoms situated around the CF group, while the high-energy intensity is formed by σ-type anti-bonding interactions between fluorine and carbon atoms within the CF group [19]. Obviously, position and relative intensity of these features are determined by the local surrounding of the CF groups. The high intensity of the pre-edge peak in the NEXAFS F K-edge spectrum of multiwalled CNTs fluorinated with a F_2_/HF mixture at 420 °C is likely due to two-sided fluorination of the shells under such harsh conditions [30].

Nine models with different quantity and distribution of fluorine atoms (seven of them are shown in Figure 4c) on the outer surface of a CNT were taken for simulation of the NEXAFS F K-edge spectra. A single F atom (model I), a pair of fluorine neighbors (model II), and a –CF_3_ group (model III) have been attached to the convex surface segment of a CNT. The latter species was considered since the plasma of CF_4_ produces CF_3_ radicals, which may bind to the carbon surface [31]. Four F atoms formed an armchair (model IV) or zigzag (model V) chain or were in (1,2) position (model VI) or (1,4) position relative to each other. We also constructed a pattern with the alternation of C=C and CF–CF bonds and a CF region of six F neighbors on one tube side (model VII). A shift required for the alignment of the theoretical spectra to the experimental energy scale was evaluated by comparing the spectrum calculated for an outer central fluorine atom of a CNT segment fluorinated on both sides (Figure S1, Supporting Information File 1) with the NEXAFS F K-edge spectrum for fully fluorinated graphite measured under the same conditions as the spectra of fluorinated DWCNTs. Theoretical F K-edge spectra showed a strong dependence of the spectral shape on the distribution of fluorine atoms (Figure S2, Supporting Information File 1).

Figure 4b presents theoretical F K-edge spectra, which in our view best fit the obtained experimental spectra. The choice was made by considering the number of peaks at energies below the σ*-edge, the distances between these peaks and their relative intensities. The σ*-edges in the calculated spectra are the least intense peaks, the energies of which are larger in the experimental spectra. This is due to limitation of the (*Z* + 1) approach used for the simulation of NEXAFS spectra, which basically reproduces well only the peaks above the absorption edge [32].

The spectrum of DWCNTs fluorinated by F_2_ well agrees with the spectrum calculated for fluorine atoms, which form a dense cluster in model VI. However, to reproduce a width of the main peak F in the NEXAFS spectrum, contributions from other fluorine patterns such as, for example, that in model VII would be helpful. It is interesting that the spectrum for model VII is similar to the spectrum calculated for CNT surfaces fluorinated on both sides (Figure S1, Supporting Information File 1). Both theoretical spectra are dominated by a single peak at lower energies than the σ*-edge and this shape is characteristic for the NEXAFS F K-edge of fully fluorinated graphite (CF)*_n_*. A decrease of the relative intensity of this peak in the spectrum of partially fluorinated graphite (C_2.5_F)*_n_* was related to a coexistence of sp^2^- and sp^3^-hybridized carbon atoms [33]. That spectrum almost coincides with the spectrum of the F_2_-fluorinated DWCNTs. Thus, we conclude the formation of small CF regions with fluorine atoms located on one or two sides of the nanotube shell when DWCNTs are fluorinated by elemental fluorine at elevated temperature.

The spectrum of DWCNTs fluorinated with BrF_3_ is in good correspondence with the spectrum calculated for the four-atom armchair chain (model IV). At energies below the edge, the spectrum of this model has two peaks, the separation and relative intensity of which agree with those for the peaks D and E in the experimental spectrum. The correspondence could be improved by taking into account other fluorine patterns such as, for example, that in model V.

Regarding the DWCNTs fluorinated with CF_4_ plasma, we were not able to find an appropriate model, of which the spectrum would suit to the experimental F K-edge spectrum. Possibly, the method produces many different fluorine distributions without any dominant pattern. We speculate that a superposition of the spectra for a single F atom (model I), for a pair of fluorine atoms in the (1,2) position (model II), and for a –CF_3_ group could give a correlation with the experiment. Actually, summing these spectra in a ratio of 2:1:1 gives a profile (top curve in Figure 4b, left part), which well reproduces the spectral features at energies below the σ*-edge. The lack of –CF_3_ groups in the XPS C 1s spectrum (Figure 3, curve 2) might be related to a substantial difference between the ionization cross-sections of C 1s electrons of bare carbon atoms and of fluorinated carbon atoms when they are excited at an energy close to the ionization threshold. The cross-section decrease for the latter kind of electrons could results in low XPS intensities, especially for –CF_2_ and –CF_3_ groups. Actually, these species were detected in the spectrum measured at 1486.6 eV (Figure S3, Supporting Information File 1). A similar behavior, particularly, a growth of the CF peak intensity with an increase of the excitation energy has been previously observed in the XPS C 1s spectra of fluorinated SWCNTs [34].

**Figure 5:**
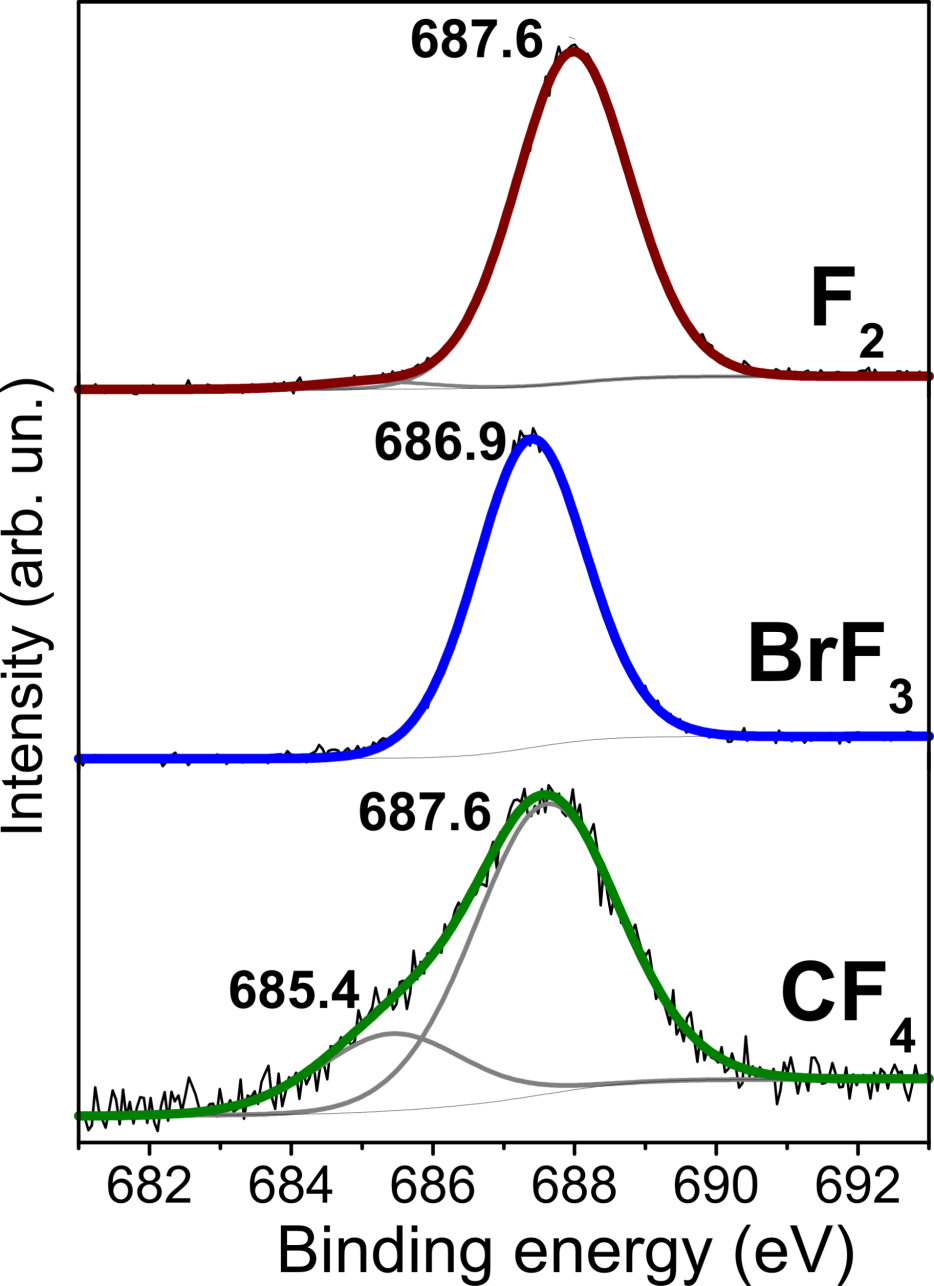
XPS F 1s spectra of DWCNTs fluorinated with CF_4_ plasma, BrF_3_, and F_2_.

The realization of different fluorination patterns on the DWCNT surface through the CF_4_ plasma technique is confirmed by XPS data of the F 1s levels (Figure 5). The spectrum of this sample has two components, while the F 1s spectra of the two other samples are presented by symmetric single peaks. Moreover, a larger width of the main component in the former spectrum is indicative of more fluorine bonding configurations in the plasma-fluorinated DWCNTs. The component at ca. 685.5 eV is often observed in the XPS F 1s spectra of plasma-fluorinated CNTs [13,35–36] and assigned to semi-ionic C–F bonds. We attribute this binding energy to fluorine atoms very distant from other fluorine atoms. Actually, the quantum-chemical calculations of fluorinated graphene models have revealed a decrease of the F 1s level energy with a C–F bond elongation [37]. The predicted shift of the F 1s level of single fluorine relative to that of the (1,2) fluorine pair is ca. 2 eV. This suits well the distance between the components in the F 1s spectrum of plasma-fluorinated DWCNTs and two fluorination patterns chosen from the modelling of NEXAFS F K-edge.

Despite difference of almost 100% in fluorine content between the DWCNT samples fluorinated with CF_4_ plasma and F_2_ (Table 1), the main peaks in their XPS F 1s spectra have the same energy (Figure 5). The position of the F 1s peak in the spectrum of the DWCNTs fluorinated with BrF_3_ is shifted to a lower binding energy by about 0.7 eV. In incompletely fluorinated graphitic materials, fluorine may interact with the electron density of the next neighboring bare carbon atoms, which causes an increase of the polarization of the C–F bond [38] and, consequently, a decrease of the F 1s electron binding energy. This is in line with the hyperconjugation mechanism involving interactions of electron density from C–F bonds with the π-electron system of graphene areas [39]. Hence, the lower binding energy of the F 1s peak in the spectrum of BrF_3_-fluorinated DWCNTs supports the chosen preferable model of short CF chains at the nanotube surface. In such chains, fluorine atoms interact with two or three bare carbon atoms.

**Figure 6:**
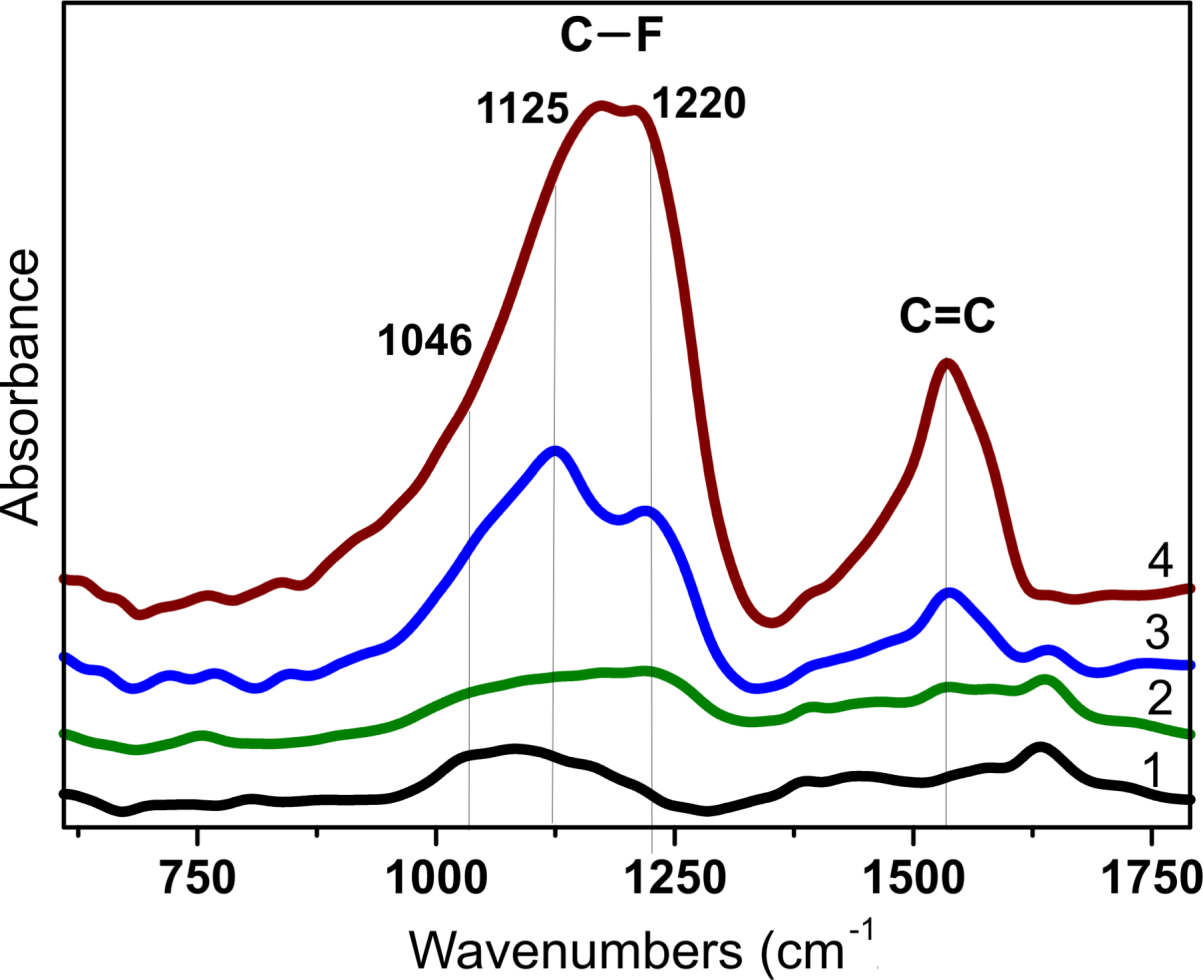
IR spectra of pristine DWCNTs (1) and DWCNTs fluorinated with CF_4_ plasma (2), BrF_3_ (3), and F_2_ (4).

FTIR spectra of the fluorinated samples confirm the difference of the dominating fluorine bonding for DWCNTs treated with the three different fluorination techniques (Figure 6). In the range of C–F stretching vibrations, the FTIR spectrum of the F_2_-fluorinated sample is dominated by a doublet peak split at 1170 and 1210 cm^−1^, whereas the BrF_3_-fluorinated sample shows instead two more separated peaks at 1125 and 1220 cm^−1^. Additionally, both spectra have a prominent shoulder at ca. 1046 cm^−1^. The spectrum of plasma-fluorinated DWCNTs exhibits the lowest intensity of C–F bond vibrations, which is likely due to the preferred fluorination of DWCNT ropes as it follows from Raman scattering and TEM data. Moreover, the exposure of DWCNTs to CF_4_ plasma has almost no effect on the absorption of the carbon lattice, while after treatment with F_2_ and BrF_3_ the intensity of the band at 1535 cm^−1^ strongly increases. This change is caused by a disruption of the uniformity of the π-system due to attachment of fluorine atoms to the CNT surface [40].

The appearance of a set of C–F absorption bands in the FTIR spectra reflects a co-existence of various types of bonds in samples. Chamssedine et al. identified three types of C–F bonding for fluorinated SWCNTs with vibration frequencies at 1220, 1100, and 1050 cm^−1^ [41]. The weakening of a covalent bond in this series was explained by a hyperconjugation with the π-electron system. Asanov et al. selected four bands for the fluorinated graphite spectrum, which were assigned to vibrations of a CF group surrounded by three CF neighbors (1230 cm^−1^), two CF neighbors and one bare carbon atom (1132 cm^−1^), one CF neighbor and two bare carbon atoms (1095 cm^−1^), and three bare carbon atoms (1045 cm^−1^) [42]. Evidently, the vibration of a certain bond may change its frequency depending of the curvature and type of surface fluorination (on one side or on two sides) [43]. However, high absorption intensities around 1210 cm^−1^ in the FTIR spectrum of the F_2_-fluorinated DWCNTs support the formation of small CF regions. Such a close position of fluorine atoms can be provided by the relatively small diameter of nanotubes and/or the penetration of some fluorine atoms between the layers, as we intended from Raman scattering in the RBM region. The dominating band at 1125 cm^−1^ in the spectrum of the BrF_3_-treated sample hints to a chain-like fluorination pattern.

The different bonding behavior is consistent with our understanding of the three fluorination processes. CF_4_ fluorination is known to give reactive CF_3_, CF_2_ and F fragments, which can then bind directly with the surface [44–45]. In contrast F_2_ fluorination is expected to result in (1,2) *ortho*- or (1,4) *para*-addition, depending on the amount of HF catalyst [16]. There is little known in the literature concerning the mechanism of BrF_3_ fluorination, so we performed a series of DFT calculations to clarify this point. Importantly, we find that a decomposition of BrF_3_ over pristine graphene to give BrF_2_ and surface-bound F is endothermic (+0.25 eV) with a reaction barrier of 0.27 eV. However, F deposition from BrF_3_ to a site neighboring a pre-existing surface fluorine atom is highly exothermic (−0.46 eV) with a similarly low barrier of 0.26 eV. This suggests that fluorination from BrF_3_ will proceed systematically from pre-existing fluorinated areas in a similar way to F_2_ fluorination, rather than distributing uniformly at low density across the surface as seen for CF_4_ plasma. These fluorination models are consistent with the experimentally observed distribution and characterization of the fluorination described above.

The fact that the pattern of fluorine addition to the CNT surface is determined by the chosen fluorination technique and is less dependend on the synthesis condition is also confirmed by a comparison of the NEXAFS F K-edge spectra for DWCNT samples with different fluorine content. The spectra of the samples treated with CF_4_ plasma for 10 and 0.5 min have the same shape, while the intensity of peak A is reduced for the sample with lower fluorine content (Figure S4a, Supporting Information File 1). From the calculation results, responsible for this peak is a pair of fluorine atoms in the (1,2)-position (Figure 4c). The F K-edge spectra of the samples fluorinated using different concentrations of BrF_3_ in the reaction volume are also mainly distinguished by the intensity of the low-energy peak (Figure S4b, Supporting Information File 1). This peak D arises from a compact armchair fluorine chain (Figure 4c) and is slightly enhanced in the spectrum of the DWCNTs with higher fluorine loading. Thus, the fluorination pattern is determined by the particular reaction mechanism, which has also been shown for SWCNTs fluorinated by gaseous F_2_ or XeF_2_ [46].

## Conclusion

DWCNTs have been fluorinated using three different agents: fluorine gas at 200 °C, gaseous BrF_3_ at room temperature, and CF_4_ plasma under mild working conditions. It was found that the resultant composition and fluorination patterning of the sample depend on the fluorination method. In the case of two latter samples, Raman spectroscopy unambiguously indicated a fluorination of the outer DWCNT shell only. In the spectrum of the F_2_-treated sample, RBMs of the inner tubes were very weak and this may be a sign of fluorine penetration between the layers. XPS C 1s spectra detected that not every carbon atom of the outer shells was bonded with fluorine. The average number of bare carbon atoms surrounding a CF group progressively grows from 1 to 3 with the use of F_2_, BrF_3_, and CF_4_ plasma. These numbers are close to the C–(CF)/CF ratios in the models selected to describe the dominating fluorination patterns for each case by comparing the NEXAFS spectra measured at the F K-edge of fluorinated DWCNTs with theoretical spectra from quantum-chemical calculations. The most probable models are small compact CF areas produced from a fast F_2_ action at high temperature, and the short armchair or zigzag CF chains, which are formed from BrF_3_ at room temperature over a few days, i.e., under conditions promoting the attachment of fluorine atoms one by one. For the DWCNTs treated with CF_4_ plasma we suppose fluorination of the rope surfaces only, since the plasma deposition is directional and the sample exposure time was relatively short. This did not allow us to choose a single model well suited to all observed experimental spectroscopy data. Moreover, the XPS F 1s spectrum showed a coexistence of at least two fluorine bonding configurations and this could be single fluorine atoms, CF pairs, and –CF_3_ groups. Thus, by application of different fluorination methods it is possible to synthesize fluorinated DWCNTs with different fluorination patterns, which should in turn be distinct in electronic properties and reactivity. Similar results are expected for other closed-shell carbon structures such as single- and multi-walled CNTs, nanohorns and onion-like carbon.

## Experimental

### Materials

DWCNTs were produced by catalytic chemical vapor deposition (CCVD) using CH_4_ (18 mol %) in H_2_ at 1000 °C and an Mg_1−_*_x_*Co*_x_*O solid solution as catalyst [23]. High-resolution transmission electron microscopy (HRTEM) showed that a typical sample consists of ca. 80% DWCNTs, 20% SWCNTs, and a few triple-walled nanotubes. The diameter distribution of the DWCNTs ranged from 0.5 to 2.5 nm for the inner tubes and from 1.2 to 3.2 nm for the outer tubes. DWCNTs were purified by heating the sample in air at 450 °C for 1 h followed by treatment with concentrated HCl to dissolve metal oxides [47].

Fluorination of DWCNTs using a mixture of F_2_ and HF, produced by electrolysis of a KF·2HF melt, was conducted at 200 °C for 10 min. Fluorination with gaseous BrF_3_ was carried out at room temperature in a Teflon flask, where the sample was held over a 10 wt % solution of BrF_3_ in Br_2_ for seven days. Plasma fluorination was performed by exposing DWCNTs to a CF_4_ plasma (frequency of 13.56 MHz and power of 15 W) for 10 min at a working pressure of 0.1 Torr. The details of the synthesis are described elsewhere [21,48].

### Instrumentation

The structure of pristine and fluorinated DWCNTs was studied using TEM on a JEOL-2010 microscope and Raman scattering using a Triplemate spectrometer (excitation wavelength 488 nm). The samples for TEM examination were prepared by ultrasonic dispersion of powder suspended in ethanol on lacey carbon film grids. The nature of the surface groups was characterized by Fourier transform IR (FTIR) spectroscopy using a Nicolet 510P spectrometer.

The XPS and NEXAFS experiments were performed at the Berliner Elektronenspeicherring für Synchrotronstrahlung (BESSY) using monochromatic radiation from the Russian–German beamline. XPS C 1s spectra were measured at an energy of 350 eV with a resolution of 0.2 eV (full width at half maximum (FWHM)). As the kinetic energy varied from 35 to 50 eV the mean free path of photoelectrons was about 0.2–0.6 nm [49], allowing us to probe the electronic state of carbon mainly from the surface layers of the fluorinated CNTs. Binding energies of the fluorinated samples were calibrated to the pristine DWCNT C 1s peak at 284.5 eV. XPS F 1s spectra were recorded on a SpecsLab PHOIBOS 150 spectrometer with Al Kα (1486.6 eV) excitation. In the spectrum analysis, the background signal was subtracted by Shirley’s method. NEXAFS spectra near the F K-edges were acquired in the total-electron yield mode with a typical probing depth of a few nanometers [50]. The spectra were normalized to the primary photon current from a gold-covered grid recorded simultaneously. Before the experiments, the samples have been annealed at 70 °C for 12 h in vacuum.

### Calculations

Quantum-chemical calculations were carried out using the three-parameter hybrid functional of Becke [51] and Lee–Yang–Parr correlation functional [52] (B3LYP method) included in the Jaguar package [53]. Atomic orbitals were described by the 6-31G* basis set. The CNT surface was modeled by a segment of an armchair (12,12) tube with C_106_H_28_ composition, where hydrogen atoms saturated dangling bonds of boundary carbon atoms. Central part of the convex segment surface was decorated with fluorine atoms for modeling possible addition patterns in DWCNTs. Positions of carbon and hydrogen atoms at the segment edges were frozen during optimization, which was conducted using an analytical method to the gradient of 5·10^−4^ atomic units for atom displacements.

Theoretical NEXAFS F K-spectra were constructed within the (*Z* + 1) approximation [54], which accounts for the effect of a final core hole created in the absorption process on the spectral profile. To model a core hole, the exciting atom was replaced by the element being next in the periodic table and, in the case of fluorine, this is neon. For compensation of the extra electron, the calculating system was charged positively. Compared to the full core-hole calculations, the (*Z* + 1) approximation requires significantly less computer resources and well fits NEXAFS C K-spectra of fullerene C_60_, CNTs and their fluorinated derivatives [32,55–56]. Intensities of spectral lines were obtained by summing the squared coefficients at Ne 2p orbitals and broadened with Lorentzian functions of a width of 0.7 eV. X-ray transition energies were determined as a difference between Kohn–Sham eigenvalues of virtual molecular orbitals of a model calculated within the (*Z* + 1) approximation (excited system) and the 1s-level energy of fluorine in the ground state of that model.

An interaction of BrF_3_ with the graphene surface was studied using the DFT code AIMPRO [57–59] by fitting the charge density to plane waves with an energy cutoff of 300 Ha. Relativistic pseudopotentials generated by Hartwigsen, Goedecker and Hutter [60] were used*.* Correspondingly, 38, 44, and 28 independent Gaussian-based functions presented basis sets for carbon, bromine, and fluorine. Electronic level occupation was obtained using Fermi occupation function with *kT* = 0.04 eV. Absolute energies were converged in the self-consistency cycle to better than 10^−9^ Ha. The surface decomposition of BrF_3_ was modeled using a spin-averaged LDA for large C_128_ graphene cells (8 × 8 supercells). Migration barriers were determined using the nudged elastic band method.

## Supporting Information

Supporting Information contains the experimental NEXAFS F K-edge spectrum of graphite fluoride (CF)*_n_* in comparison with the calculated spectrum; NEXAFS F K-edge spectra plotted for different fluorine distributions on carbon nanotube surfaces; the XPS C 1s spectrum of plasma fluorinated DWCNTs measured at 1486.6 eV; NEXAFS FK-edge spectra for DWCNTs fluorinated with CF_4_ plasma for different periods as well as DWCNTs fluorinated using different concentration of BrF_3_ vapors.

File 1 Additional experimental data.
