# Supplementary material for: Effect of the fluorination technique on the surface-fluorination patterning of double-walled carbon nanotubes
Source: Beilstein J Nanotechnol. 2017 Aug 15;8:1688–98. doi: 10.3762/bjnano.8.169 (PMC5564273; doi:10.3762/bjnano.8.169)
Supplement: File 1 — Additional experimental data. [file Beilstein_J_Nanotechnol-08-1688-s001.pdf]

Supporting Information

for

Effect of the fluorination technique on the surface-fluorination patterning of double-walled carbon nanotubes

Lyubov G. Bulusheva*[,1,2], Yuliya V. Fedoseeva[1,2], Emmanuel Flahaut[3,4], Jérémy Rio[5], Christopher P. Ewels[5], Victor O. Koroteev[1,2], Gregory Van Lier[6], Denis V. Vyalikh[7,8] and Alexander V. Okotrub[1,2]

Address: [1]Nikolaev Institute of Inorganic Chemistry, SB RAS, 630090 Novosibirsk, Russia; [2]Novosibirsk State University, 630090 Novosibirsk, Russia; [3]Centre Interuniversitaire de Recherche et d'Ingenierie des Materiaux, Universite Paul-Sabatier, F-31062 Toulouse 9, France [4]CNRS, Institut Carnot Cirimat, F-31062 Toulouse, France; [5]Institut des Materiaux Jean Rouxel, CNRS-Université de Nantes, F-44322 Nantes, France; [6]Vrije Universiteit Brussel – Free University of Brussels (VUB), Pleinlaan 2, B-1050 Brussels, Belgium; [7]Donostia International Physics Center, Departamento de Fisica de Materiales and CFM-MPC UPV/EHU, 20080 San Sebastian, Spain and [8]IKERBASQUE, Basque Foundation for Science, 48011 Bilbao, Spain

Email: Lyubov G. Bulusheva* - bul@niic.nsc.ru

* Corresponding author

Additional experimental data

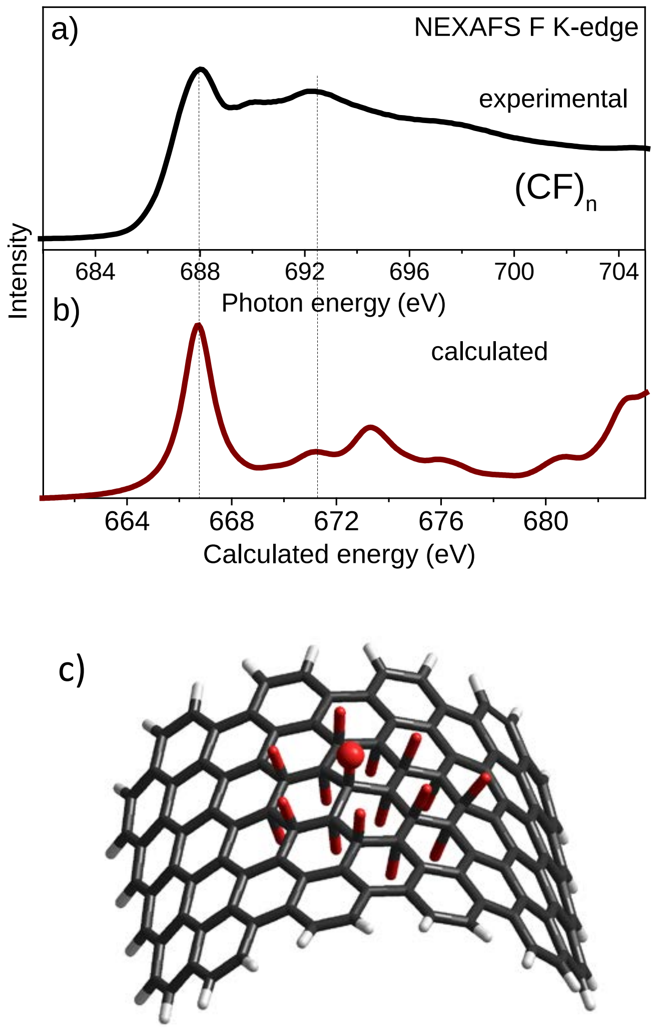

Figure S1: Comparison of experimental NEXAFS F K-edge spectrum (a) of fully fluorinated graphite $(CF)_n$ and theoretical spectrum (b) calculated for an outer central fluorine atom (shown by a ball) in a CF area on carbon tube segment with composition of $C_{106}H_{28}F_{16}$ (c). Experimental and calculated spectra were aligned by position of the first peak. The difference of 20.9 eV was used to recalculate the energy scale for the theoretical spectra presented in Figure 4b.

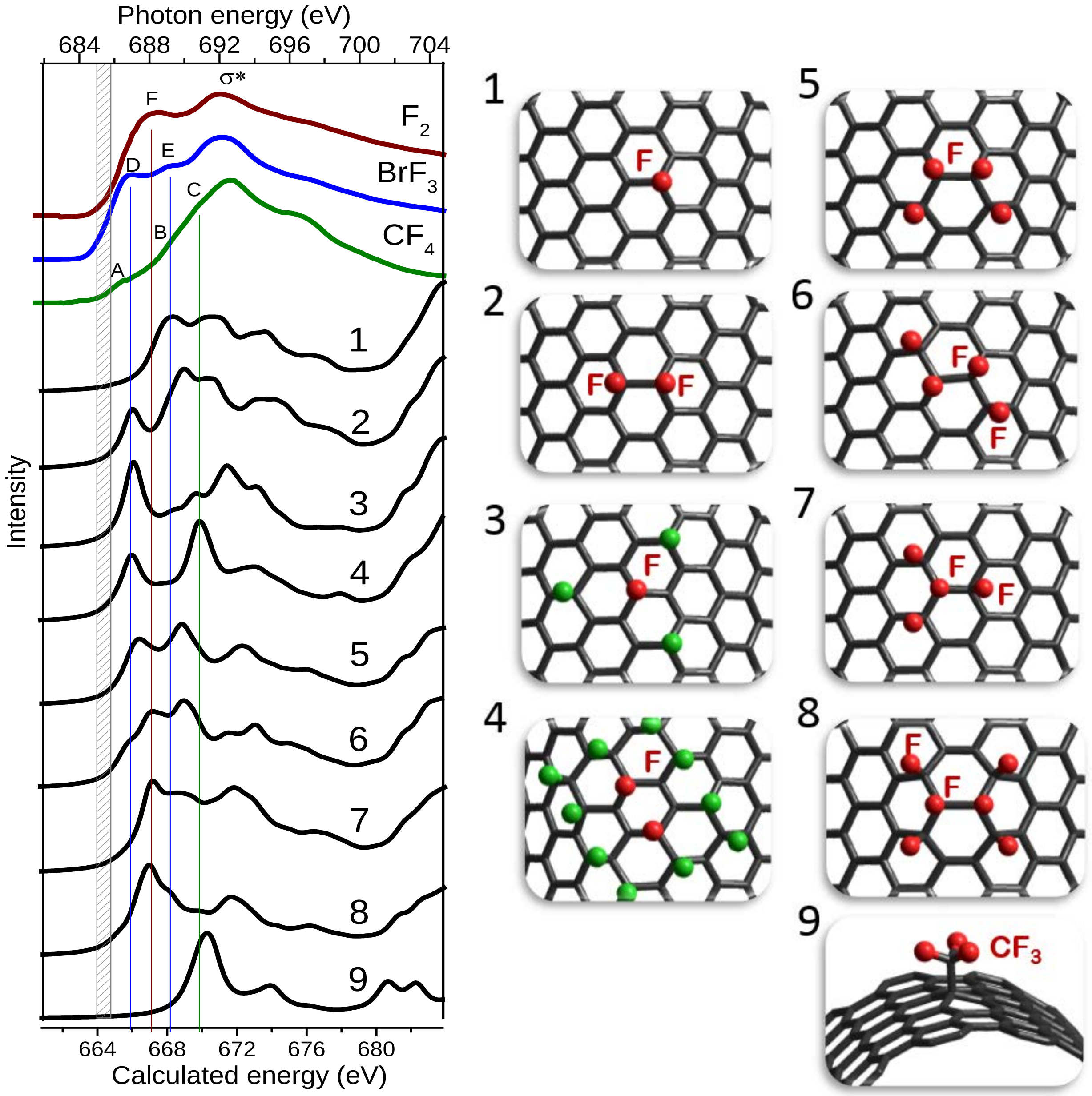

Figure S2: Comparison of NEXAFS F K-edge spectra measured for DWCNTs fluorinated using F_2, BrF_3 and CF_4 plasma with the spectra calculated for fluorine atoms (highlighted by red) attached to convex surface of carbon tube segments in different quantity and pattern. We considered the models with a single F atom (structure 1), a pair of F neighbors located in (1,2) position (structure 2), four F atoms in (1,4) positions (structure 3), alternating C=C and CF–CF bonds (structure 4), four-atom armchair (structure 5) and zigzag (structure 6) chains, CF clusters consisting from four F atoms (structure 7) and six F atoms (structure 8), and $–CF_3$ group (structure 9).

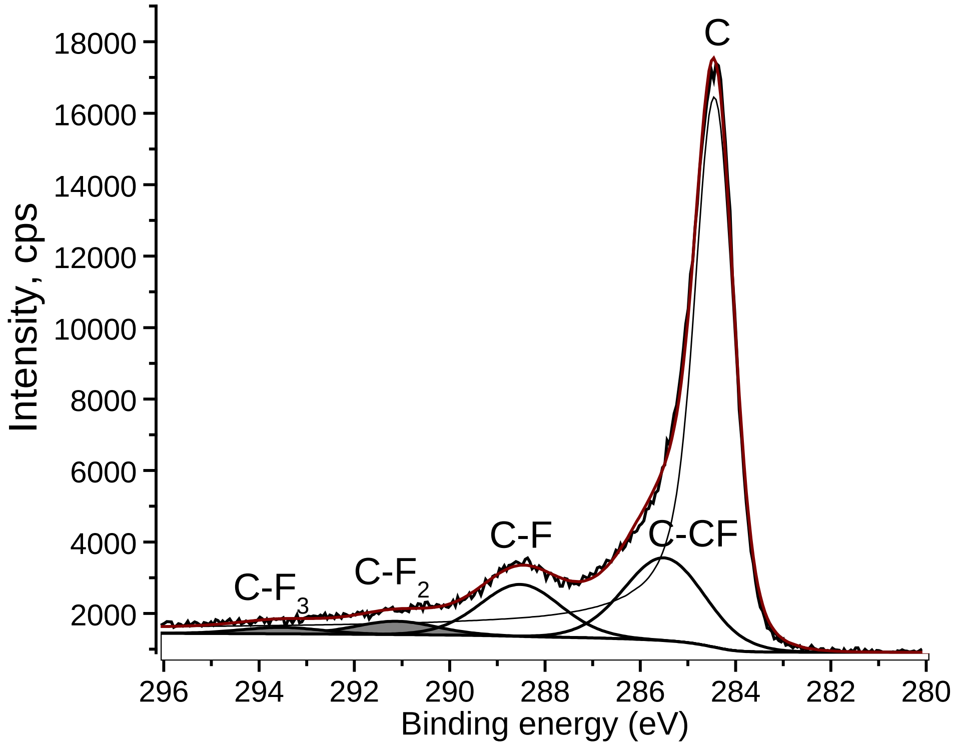

Figure S3: XPS C 1s spectrum for CF_4 plasma-fluorinated DWCNTs measured at an excitation energy of 1486.6 eV.

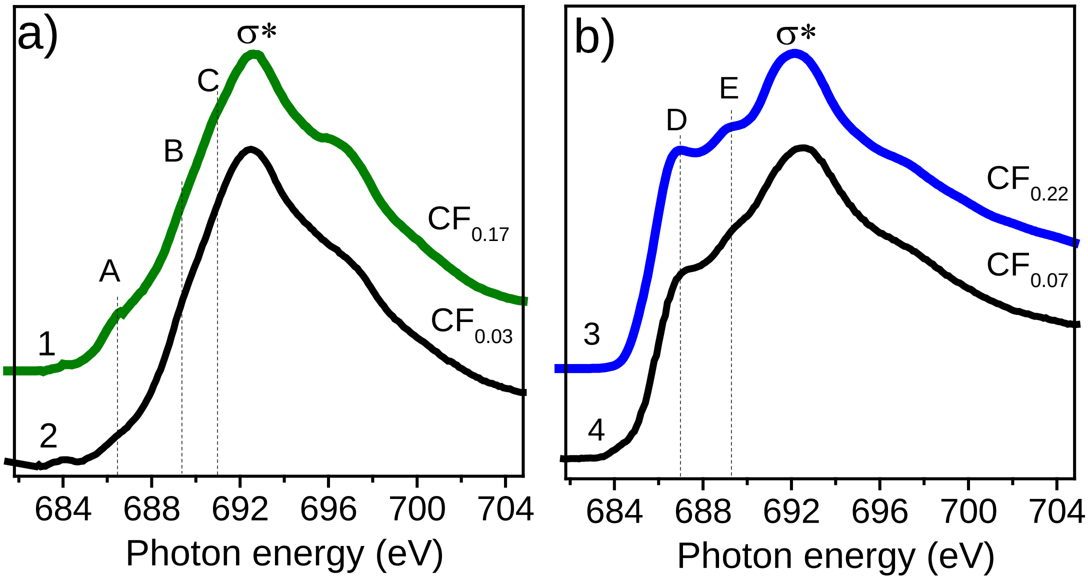

Figure S4: Experimental NEXAFS F K-edge spectra for (a) CF_4 rf plasma-fluorinated DWCNTs for 10 min (1) and 0.5 min (2), and (b) BrF_3 fluorinated DWCNTs using 10 wt % (3) and 3 wt % (4) BrF_3 in Br_2. The stoichiometry of fluorinated DWCNTs was estimated from XPS data.
